# Diseases of the Nose and Throat

**Published:** 1893-08

**Authors:** 


Diseases of the Nose and Throat. A Text-book for Students and Practitioners. By Horace F. Ivins, M. D., with 129 illustrations, including 18 colored figures: Philadelphia (1914 Cherry Street), and London: The F. A. Davis Co., Publishers, 1893. Price, cloth, $4 net.

This is a volume of 500 pages upon the treatment of the nose and throat by a physician practicing Homœopathy. The treatment is mainly operative interference, though indications for remedies are also given. The book is divided into three parts. Part I, the nose, contains an accurate description of the anatomy of the internal nose, illustrated by elegant views, one at least being a photogravure. These views are very successful, as any one will know who has sought, with difficulty, to get clear ideas of this part of the human form from drawings. All the various phases of diseases affecting the nose are treated in separate chapters with excellent descriptions of apparatus and instruments, abundantly illustrated.

Part II treats of the pharynx and its diseases, upon the same plan as the description of the nose, and part III treats of the larynx in the same way. A very interesting account of the production of vocalization is also given. The work is enriched by colored drawings of the appearances seen in the examining mirror. The strictly homœopathic resources of treatment are, of course, disappointing to an enthusiastic homœopathist.

